# Withdrawal: Ca^2+^/calmodulin-dependent protein kinase II promotes cell cycle progression by directly activating MEK1 and subsequently modulating p27 phosphorylation

**DOI:** 10.1016/j.jbc.2021.100766

**Published:** 2021-05-23

**Authors:** Nan Li, Chunmei Wang, Yanan Wu, Xingguang Liu, Xuetao Cao

VOLUME 284 (2009) PAGES 3021–3027

This article has been withdrawn by the authors. The Journal states that Fig. 5*B* presented many similar repeating features in the background of the top two left immunoblots. The authors state that they disagree with the Journal’s findings. The authors state that this study was conducted more than 10 years ago. The age of the work and the lack of availability of data storage makes it difficult for the authors to retrieve the requested raw data. The authors’ offer to replace the images in question with those from another experiment with the same conditions was declined by the Journal. The authors state that the above issues do not affect the interpretation of the data nor conclusions of this research study. The authors affirm that phosphorylation of p27 occurs by activation of the MEK/ERK cascade reported in the article has been confirmed by other independent studies, and they have indicated that they plan to resubmit their findings elsewhere for publication.

